# The Change of Life in Health and Disease

**Published:** 1871-04

**Authors:** 


### Book Notices.

The Change of Life in Health and Disease. A Practical Treatise on the Nervous and Other Affections Incidental to Women at the Decline of Life. By Edward John Tilt, M.D. From the third London edition. Lindsay & Blakiston, Publishers, Philadelphia. For sale by S. C. Griggs & Co., Chicago.

This is a volume of 287 pages, comprising twelve chapters. The first five are an introductory, one on the physiology of the change of life, one on its pathology, one on its therapeutics, and one on its hygienics. Then follow chapters which treat consecutively of the diseases of the digestive organs, and of the skin; the tenth treats of the diseases of the ganlionic nervous system, and the eleventh, of the cerebro-spinal affections; and the concluding chapter is miscellaneous.

Additional value is given to the volume by numerous interesting tables, which exhibit various physiological and pathological facts in a clear and definite manner. As a work containing a large amount of valuable practical information upon a subject not very generally understood, we cordially recommend it to the profession.

